# Biphasic Somatic A-Type K^+^ Channel Downregulation Mediates Intrinsic Plasticity in Hippocampal CA1 Pyramidal Neurons

**DOI:** 10.1371/journal.pone.0006549

**Published:** 2009-08-07

**Authors:** Sung-Cherl Jung, Dax A. Hoffman

**Affiliations:** Molecular Neurophysiology and Biophysics Unit, Laboratory of Cellular and Synaptic Neurophysiology (LCSN), NICHD, National Institutes of Health (NIH), Bethesda, Maryland, United States of America; Vrije Universiteit Amsterdam, Netherlands

## Abstract

Since its original description, the induction of synaptic long-term potentiation (LTP) has been known to be accompanied by a lasting increase in the intrinsic excitability (intrinsic plasticity) of hippocampal neurons. Recent evidence shows that dendritic excitability can be enhanced by an activity-dependent decrease in the activity of A-type K^+^ channels. In the present manuscript, we examined the role of A-type K^+^ channels in regulating intrinsic excitability of CA1 pyramidal neurons of the hippocampus after synapse-specific LTP induction. In electrophysiological recordings we found that LTP induced a potentiation of excitability which was accompanied by a two-phased change in A-type K^+^ channel activity recorded in nucleated patches from organotypic slices of rat hippocampus. Induction of LTP resulted in an immediate but short lasting hyperpolarization of the voltage-dependence of steady-state A-type K^+^ channel inactivation along with a progressive, long-lasting decrease in peak A-current density. Blocking clathrin-mediated endocytosis prevented the A-current decrease and most measures of intrinsic plasticity. These results suggest that two temporally distinct but overlapping mechanisms of A-channel downregulation together contribute to the plasticity of intrinsic excitability. Finally we show that intrinsic plasticity resulted in a global enhancement of EPSP-spike coupling.

## Introduction

For several decades, synaptic plasticity has been considered the best candidate mechanism for the formation and storage of memories. However, efficacy in driving a neuron to fire an action potential (AP) is dependent not only on the size, but also the location and timing of synaptic input, which is subsequently shaped by types and distributions of voltage- and calcium-gated conductances in dendrites. A number of studies, including the original description of long-term potentiation (LTP) [1], have reported that the induction of synaptic plasticity is accompanied by changes in the intrinsic excitability of the neuron, indicating a potential concurrent change in voltage-gated channel activity [2], [3]. Ac tivity-dependent regulation of intrinsic excitability has been observed in several invertebrate and vertebrate preparations [4], [5] and is induced by learning [6]. Changes in voltage-gated channel expression and/or function could mediate these changes in excitably after LTP (intrinsic plasticity). If intrinsic plasticity does act as a memory-storage mechanism [4], it is essential to understand how voltage- and/or calcium-gated channels are modulated and how this plasticity in their function contributes to learning and memory.

With a subthreshold activation range, A-type K^+^ channels are rapidly activated upon depolarization and so can influence AP onset time, threshold, and inter-spike intervals [7]. More recently, a number of other functions for A-type K^+^ channels have been described, including aiding in AP repolarization, frequency dependent AP broadening, controlling action potential back propagation into dendrites [8], [9], regulating the induction of synaptic plasticity [10] and in determining timing of synaptic inputs [11]. We have shown that one particular voltage-gated potassium subunit (Kv4.2), controls the initiation, duration and backpropagation of action potentials in CA1 pyramidal neurons from hippocampal organotypic slice cultures [8]. Moreover, surface membrane expression of Kv4.2 channels is regulated in an activity- and NMDAR-dependent manner [12]. Trafficking of voltage-gated channels therefore provides another way neurons may dynamically regulate excitability in addition to modulation of channel kinetic properties.

The search for the mechanism of intrinsic plasticity has provided evidence for changes in the voltage-dependent properties of a number of ion channels after LTP induction [2], [3], [13]–[17]. In CA1 dendrites, Frick et al. have shown that LTP induction results in a leftward shift in the voltage-dependence of steady-state inactivation curve of A-type K^+^ currents in acute hippocampal slices from adult rats [18]. This shift has the effect of increasing local dendritic excitability, enhancing action potential back propagation. However, LTP also causes a decrease of AP firing threshold, a global phenomenon [19].

We show here that LTP induction results in a rapid, long-lasting increase in the intrinsic excitability of CA1 pyramidal neurons from organotypic slice cultures, including a change in initial AP threshold. This LTP-induced increase in excitability was accompanied by a two-phased decrease in A-current activity. Upon LTP induction, we observed in nucleated patches an immediate but transient (∼10–20 min) hyperpolarized shift in the voltage-dependence of steady-state inactivation for A-type K^+^ currents. This shift was accompanied by a progressive, long-term decrease in peak A-type K^+^ current amplitude that outlasts the observed A-channel inactivation curve shift. Blocking clathrin-mediated endocytosis by intracellular delivery of a dynamin-based inhibitory peptide completely prevented the late expression of LTP and most measures of intrinsic plasticity. Finally we show that this type of plasticity acts globally, with its induction enhancing the synaptic efficacy of un-potentiated synapses. These results indicate that voltage-dependent A-type channels crucially contribute to the enhancement of EPSP-spike coupling with implications for the memory-storage capacity of the hippocampus.

## Results

### Long-lasting enhancement of intrinsic excitability after LTP induction

**Figure 1:**
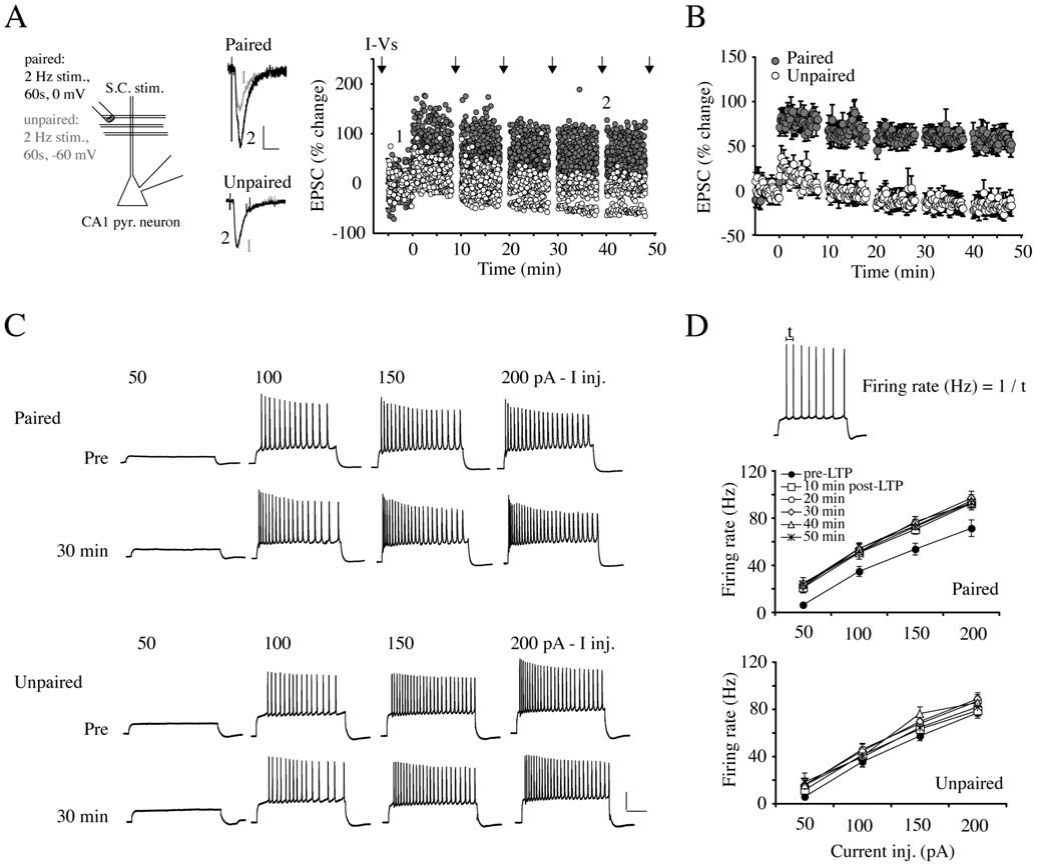
Synaptic potentiation increases intrinsic excitability. A. Experimental schema for conditioning stimulation to induce synaptic potentiation. Synaptic LTP was induced by 2 Hz stimulation of the Schaffer collateral (S.C.) pathway for 1 min paired with depolarization to a 0 mV holding potential (“Paired”). For control, the same stimulating protocol was delivered without depolarization (“Unpaired”). The right panel shows synaptic responses of individual neurons before and after conditioning stimulation. Synaptic potentiation was measured as the amplitude of EPSCs triggered by S.C. test stimulation (0.1 Hz) before and after conditioning stimulation. Inset traces are EPSCs recorded at the indicated time. Scale bars: 50 pA, 20 ms. To measure intrinsic excitability, a series of current injections (+50 to +200 pA in 50 pA increments) was delivered to elicit APs (“I-Vs”, arrows) in I-clamp mode every 10 min before and after LTP. B. Pooled LTP data from “paired” and “unpaired” neurons. After a 5 min stable baseline, significant potentiation of synaptic strength was observed in neurons receiving paired stimulation while unpaired neurons showed no change in EPSC amplitude. Error bars represent SEM. C. Examples of AP firing patterns triggered by current injections before and 30 min after conditioning stimulation. Current injections over 100 pA generally fired APs. Firing rates increased after conditioning stimulation in paired but not in unpaired neurons. Scale bars: 20 mV, 200 ms. D. Firing rate was calculated by measuring the interval between first and second AP. Firing rates were significantly increased for all current injections in neurons that received paired stimulation. No change in initial AP frequency was observed in neurons received unpaired stimulation. Error bars represent SEM.

**Figure 2:**
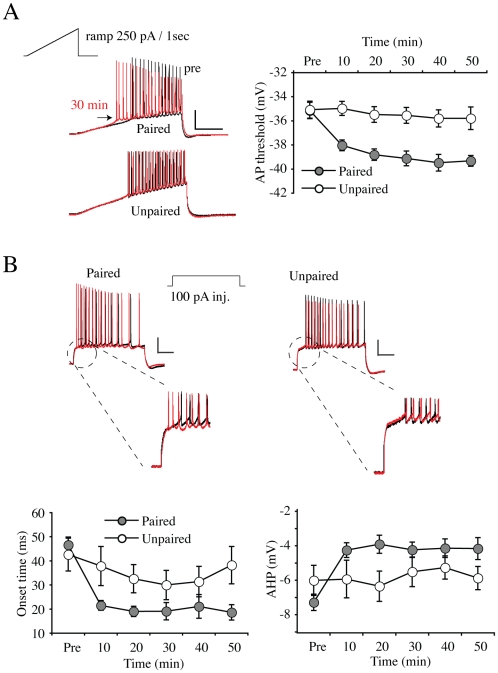
Synaptic potentiation reduces AP onset, threshold and AHPs. A. Current ramps (250 pA/s) were used to determine AP threshold. Examples of APs traces recorded before (black) and 30 min (red) after conditioning stimulation are given for paired and unpaired conditions. Scale bars: 40 mV, 200 ms. (right panel) Pooled data. The threshold to fire the first AP was lowered initially after LTP induction and this decrease persisted throughout the recording episode. B. Example traces upon +100 pA current injection before (black) and 30 min (red) after conditioning stimulations. AP onset time and after-hyperpolarizing potential (AHP) were measured before and every 10 min after conditioning stimulation. Scale bars: 20 mV, 200 ms. (left lower panel) Pooled onset time data. Onset time was significantly decreased in paired neurons, while unpaired neurons did not show any significant decrease. As with AP threshold, the onset time changes occurred initially after LTP induction and persisted throughout the recording. (right lower panel) Pooled AHP data. AHP also persistently decreased only in paired neurons. Error bars represent SEM.

Having recently described the activity-dependent trafficking of the A-type K^+^ channel subunit Kv4.2 in hippocampal neurons [12], we hypothesized that A-channel internalization contributes to intrinsic excitability changes observed in CA1 hippocampal pyramidal neurons after synaptic LTP induction [1], [19]. LTP was induced by pairing low frequency stimulation (2 Hz, 0.1 ms duration, 1 min) of the Schaffer-Collateral pathway with depolarization to 0 mV. We and others have previously found LTP induced in these experimental conditions to be NMDAR- and CaMKII- dependent [20], [21]. We monitored LTP expression by measuring EPSC amplitude change in voltage-clamp mode at −60 mV (Figure 1A and B, “Paired”, 64.33±12.13% increase in EPSC amplitude 40 min after pairing, n = 8, p = 0.007 compared with “Unpaired”). The same conditioning stimulation protocol without depolarization (−60 mV holding potential) did not result in synaptic LTP (Figure 1A–B, “Unpaired”, n = 6, −11.95±9.51% potentiation, 40 min after pairing, p = 0.315). We measured changes in intrinsic excitability by monitoring AP firing patterns induced by a series of step and ramp current injections in whole-cell current clamp recordings (Figure 1 and 2). This firing profile was observed before and every ten minutes after LTP induction. In both groups (Paired and Unpaired), current injections greater than 100 pA were generally required to initiate APs before LTP induction. In some cases APs were observed with 50 pA current injections (paired n = 4, unpaired n = 3). After LTP pairing stimulation, the number of cells firing with 50 pA current injections increased (10 min post-LTP, paired = 6, unpaired = 4). We measured AP firing rate (Hz) between the first and second AP (first interspike interval). Increased firing frequency was observed 10 min after LTP induction and maintained throughout the recordings (Figure 1C and D, “Paired”, +100 pA injection: pre = 38.84±4.27 Hz, 40 min = 54.30±4.79 Hz, p = 0.013) in paired neurons. However, the increased firing rate was not observed in unpaired recordings (Figure 1C and D, “Unpaired”, +100 pA injection: pre = 35.62±4.23 Hz, 40 min = 40.25±6.57 Hz, p = 0.407). These data establish that intrinsic plasticity is observed in CA1 neurons of organotypic slice cultures after depolarization-pairing induced LTP.

Previously we found the A-type K^+^ channel subunit Kv4.2 to influence a number of parameters of excitability in CA1 neurons, including changes in AP onset time, threshold and the magnitude of after-hyperpolarization potentials (AHPs) [8]. In Figure 2, we analyzed these parameters before and after LTP induction. As in our previous study, action potential threshold was determined by applying the ramp currents (250 pA/s, Figure 2A). LTP induction led to an initial decrease in AP threshold which remained throughout the 50 min recording period and which was not induced in unpaired neurons (Figure 2A; “Paired”, pre = −35.14±0.67, 40 min = −39.47±0.69 mV, p<0.001: “Unpaired”, pre = −35.07±0.71, 40 min = −35.79±0.72 mV, p = 0.745). This result is compatible with a downregulation of Kv4.2 activity [8]. A number of other measures of excitability regulated by Kv4.2 expression level were also altered after LTP induction in a manner consistent with a decrease in Kv4.2 function. The onset time of the first AP fired in response to a step current injection of +100 pA was significantly decreased initially after LTP induction in paired neurons (Figure 2B). This decrease persisted throughout the recording period (“Paired”, pre = 46.61±3.29, 40 min = 21.11±4.94 ms, p = 0.010). We also observed a decrease in onset time in recordings from unpaired neurons although it did not reach statistical significance (“Unpaired”, pre = 42.62±6.83, 40 min = 31.41±10.03 ms, p = 0.287). Similarly, we observed an initial and sustained decrease in the first AHP, measured as the voltage difference between AP threshold and the peak after- hyperpolarization voltage, in recordings from paired but not unpaired neurons (Figure 2B; “Paired”, pre = −7.28±0.47, 40 min = −4.14±0.54 mV, p<0.001; “Unpaired”, pre = −6.01±0.87, 40 min = −5.26±0.68 mV, p = 0.247).

### Biphasic regulation of somatic I_A_ after LTP induction

**Figure 3:**
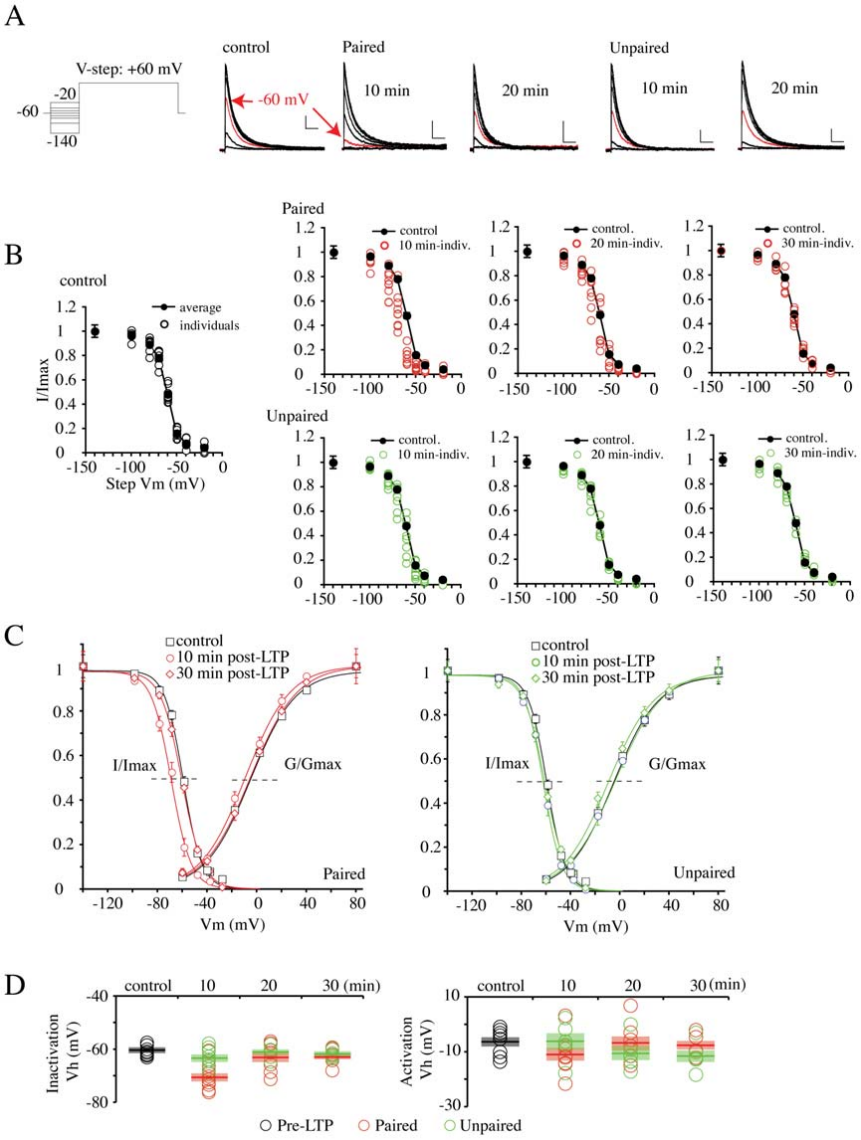
LTP modulates steady-state inactivation of A-type K^+^ currents. A. Examples of A-type currents recorded at +60 mV from a variety of holding potentials, before and 10 and 20 min after conditioning stimulation in paired and unpaired neurons. The red trace in each set is the A-current elicited from the resting membrane potential in our current-clamp recordings (−60 mV). Scale bars, 200 pA, 50 ms. The A-current available for activation at −60 mV was significantly decreased only in the neurons receiving paired stimulation 10 min after conditioning stimulation (a red arrow). However, no change was found 20 min after LTP in paired neurons or at any time in unpaired neurons. B. All steady-state inactivation data for individual recordings at 10, 20 and 30 min after conditioning stimulation for neurons receiving paired (red circles) and unpaired (green circles) stimulation is plotted and compared to the average steady-state inactivation curve measured before LTP stimulation. C. Averaged activation and inactivation properties before and after conditioning stimulation. The hyperpolarizing shift of the A-current steady-state inactivation curve observed in paired neurons 10 min after conditioning stimulation was not observed in paired neurons at later times 20 or 30 min (red curves) or for neurons receiving unpaired stimulation (green curves). No significant change in the voltage-dependence of activation was found for either group at any time point. D. Voltage for half-maximal steady-state inactivation (left panel) and activation (right panel) are plotted for each group (red-paired, green-unpaired) before (control) and 10, 20 and 30 min after conditioning stimulation. Bars indicate the mean value of each group and semitransparent boxes represent SEM.

A previous report found LTP induction to induce a hyperpolarizing shift in the steady-state inactivation curve for A-channels in cell-attached patches in CA1 dendrites, leading to enhanced action potential backpropagation [18]. Such a shift could account for the enhanced excitability observed in Figures 1 and 2. To investigate changes in A-channel properties after LTP induction, we pulled nucleated patches from CA1 neurons before (“control”, n = 9), as well as 10 (paired, n = 7, unpaired, n = 8), 20 (paired, n = 7, unpaired = 6) and 30 min (paired, n = 7, unpaired = 5) after delivering paired and unpaired stimulation protocols in organotypic slices (Figure S1). Figure 3 shows LTP induction to result in a rapid but short-lived hyperpolarization of the voltage-dependence of steady-state inactivation for I_A_. This ∼10 mV curve shift was observed in neurons 10 min after receiving paired stimulation (Figure 3; V_h,_ control = −60.7±0.6 mV; “Paired”, 10 min = −70.2±1.5 mV, p<0.001). The hyperpolarizing shift after pairing stimulation was not observed in unpaired neurons, and neither condition induced a significant change in the voltage dependence of activation (Figure 3C). Given the steep voltage-dependence of inactivation, the curve shift results in a severe reduction (62.3%, Figure 3B and C) of I_A_ near resting potentials (∼−60 mV, Figure 3A, red traces), which would be expected to increase CA1 excitability. However, this shift shown in paired neurons after 10 min cannot fully explain our intrinsic plasticity results; while enhanced excitability after LTP induction persists throughout the 50 min recording period (Figures 1 and 2), the inactivation curve reverted back to the pre-LTP state by 20 min after conditioning stimulation (Figure 3C and D; V_h_, 20 min = −63.2±1.9, p = 0.280, 30 min = −62.6±1.2 mV, p = 0.216, compared with “control”).

**Figure 4:**
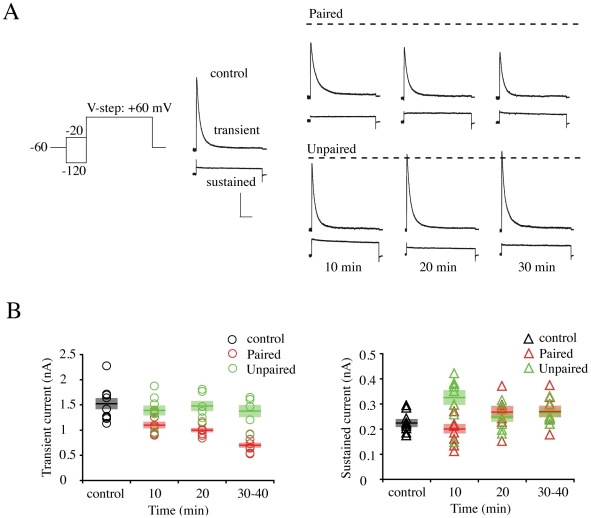
Synaptic LTP decreases peak A-current density. A. Total A-current was acquired by subtracting the sustained current (obtained using a prepulse to inactivate I_A_ as shown in the voltage protocol) from total current. Each trace is an average of three sweeps. In paired neurons showing potentiation of synaptic transmission, peak I_A_ progressively decreased with time after LTP induction. Dashed line indicates control amplitude. Scale bars 500 pA, 100 ms. B. Summarized changes of peak amplitude of transient (left panel) and sustained (right panel) currents. Significant and progressive reduction of peak transient current was observed in paired neurons (red circles and bars) after conditioning stimulation without any changes of sustained currents. No change in peak A-current was observed in neurons not exhibiting a potentiation in synaptic strength (unpaired, green circles and bars). A significant increase in sustained current amplitude was observed in unpaired neurons 10 min after conditioning but this change was not lasting. Bars indicate the mean value of each group and semitransparent boxes represent SEM.

Our previous report demonstrating the activity-dependent downregulation of I_A_ in cultured neurons suggested another possible mechanism by which I_A_ could contribute to intrinsic plasticity[12]. Here, we measured peak I_A_ in nucleated patches for voltage steps from −120 mV to +60 mV (Figure 4A). Peak I_A_ before conditioning stimulation averaged 1.5 nA (Figure 4A and B, control = 1.52±0.11 nA). In neurons receiving unpaired stimulation, which did not show synaptic potentiation, no reduction of peak I_A_ was observed (Figure 4A and B; “Unpaired”, 30 min = 1.37±0.12 nA, p = 0.441, compared with “control”). However, a significant and time-dependent decrement of I_A_ peak was observed in neurons exhibiting synaptic potentiation after paired stimulation (Figure 4A and B). Peak I_A_ decreased by about 30%, 10 min after LTP induction (“Paired”, 10 min = 1.10±0.06 nA, p = 0.009) and by 30 min, less than half the “control” level of I_A_ remained (“Paired”, 30 min = 0.69±0.05 nA, p<0.001). No change, at any time point was observed after LTP induction for the sustained, non-inactivating component of the total outward current (Figure 4). However, a small but significant *increase* of sustained current was observed in unpaired neurons 10 min after conditioning stimulation, compared with “control” (control = 0.23±0.01; “Unpaired”, 10 min = 0.32±0.03 nA, p = 0.016). This increase did not persist beyond the initial 10 min time point (30 min, paired = 0.27±0.02, unpaired = 0.26±0.02 nA).

These findings suggest a model whereby a biphasic reduction in I_A_ activity contributes to intrinsic excitability changes observed after LTP induction. Initially, LTP results in a rapid but short-lived decrease in I_A_, through a hyperpolarizing shift in the inactivation curve. A slow developing but persistent rundown in total transient current follows this temporary reduction. If the second phase occurs through activity-dependent internalization, it should be possible to block intrinsic plasticity by preventing clathrin-mediated endocytosis [12].

### Clathrin-mediated endocytosis is required for both synaptic and intrinsic plasticity

**Figure 5:**
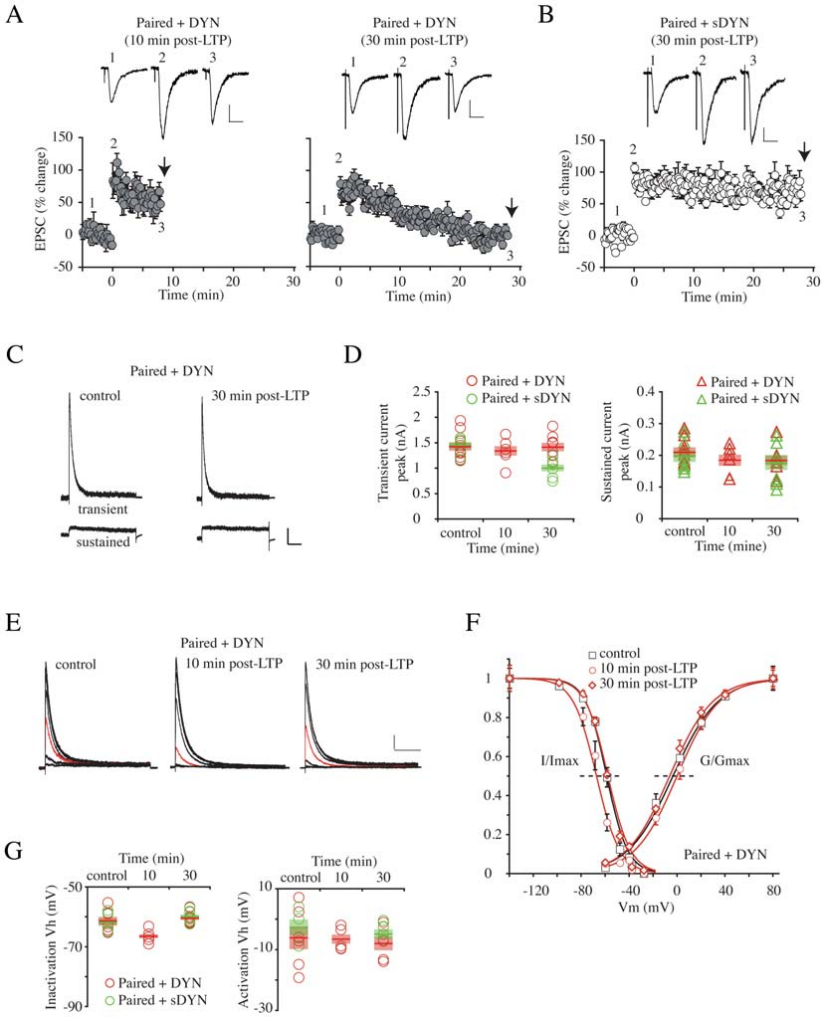
A dynamin-based inhibitory peptide (DYN) blocks LTP and the reduction of A-type K^+^ currents. In these experiments we included DYN in the patch pipette to prevent clathrin mediated endocytosis [12]. Transient and sustained current densities and voltage-dependent properties were observed in nucleated patches pulled either 10 or 30 min after LTP induction and compared with control. A. DYN added to pipette recording solution (100 µg/ml) allows for an initial potentiation 10 min after LTP induction (p<0.003 compared with control). However, potentiation of synaptic transmission was completely lost 20 min after paired stimulation in the presence of DYN. Inset traces are EPSCs recorded at the indicated time. Scale bars: 50 pA, 20 ms. Error bars represent SEM. B. Scrambled DYN (sDYN, 100 µg/ml) did not affect LTP. The arrows in A and B indicate the time when nucleated patches were formed from whole-cells. Inset traces are EPSCs recorded at the indicated time. Scale bars: 50 pA, 20 ms. Error bars represent SEM. C. Example traces of peak K^+^ currents recorded in the “Paired+DYN” group. In the presence of DYN, peak amplitude of I_A_ was not reduced after paired stimulation. Recording protocols is as in Figure 4A. Scale bars: 200 pA, 100 ms. D. Averaged peak amplitudes of transient and sustained currents after conditioning stimulations in the presence of DYN and sDYN. DYN completely blocked the decrease of I_A_ peak after paired stimulation while a significant reduction of I_A_ was still observed 30 min post-LTP in the “Paired+sDYN” group. Sustained currents did not show any changes in either group. Bars indicate the mean value and semitransparent boxes represent SEM. E. Example traces used to construct steady-state inactivation curves before and after paired stimulation in the presence of DYN. Red traces indicate I_A_ recorded at a +60 mV from a −60 mV membrane potential. Recording protocols is as in Figure 3A. Scale bars: 200 pA, 100 ms. F. Averaged inactivation and activation properties of I_A_ before and after paired stimulation in the presence of DYN. The left-shifted inactivation curve of I_A_ was still observed 10 min after LTP induction. For comparison, the results of sDYN are shown in Figure S2. Error bars represent SEM. G. Averaged changes of V_h_ (dotted lines in F) in the presence of DYN (red symbols) or sDYN (green symbols). V_h_ of steady-state inactivation showed a significant change only10 min after LTP induction, consistent with results of experiments performed without DYN. Bars indicate the mean value and semitransparent boxes represent SEM.

**Figure 6:**
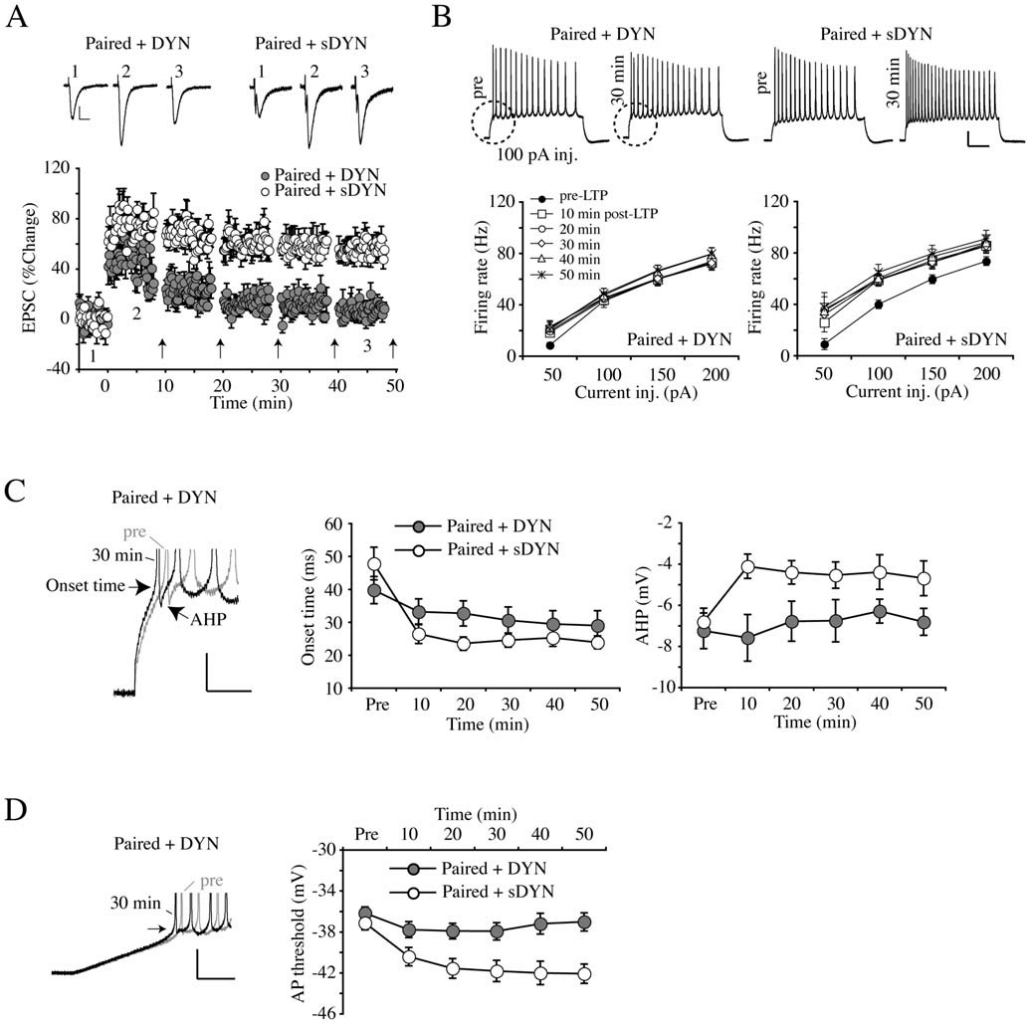
A dynamin inhibitory peptide blocks intrinsic plasticity. A. Pooled synaptic potentiation for experiments using an intracellular dynamin inhibitory peptide (DYN) and control (scrambled DYN, sDYN). Top traces are EPSCs recorded at the time indicated by the number in the pooled data plot below. Intrinsic excitability was measured every 10 min after paired stimulation. Scale bars: 50 pA, 20 ms. Error bars represent SEM. B. Change of AP firing rates after conditioning stimulations in the presence of DYN or sDYN. Upper traces show AP firing in response to a +100 pA current injection before (pre) and 30 min after LTP induction. AP firing rates were not increased by paired stimulation in the presence of DYN, while increased firing was still observed in neurons recorded with sDYN in the pipette. Portion of traces outlined in dashed circles are expanded in panel C. Scale bars: 20 mV, 200 ms. Error bars represent SEM. C. Example traces shown in the dashed circles of B were scaled up and overlapped for comparison to see the changes of onset time and AHP. DYN prevented the decrease of AHP and onset time by paired stimulation. Scale bar: 10 mV, 50 ms. Average changes in first AP onset time and AHP in the presence of DYN or sDYN are shown in the left panel and the right panel, respectively. Error bars represent SEM. D. Example traces of AP firing after injecting current ramps (250 pA/s) to measure the change of AP threshold before and after paired stimulation. DYN completely blocked the change of threshold after pairing, compared to the sDYN group. Scale bars: 20 mV, 100 ms. Error bars represent SEM.

To examine the role of activity-dependent endocytosis of A-type channels in intrinsic plasticity, we blocked the internalization of A-type K^+^ channels by including a dynamin-based peptide (DYN, 100 µg/ml) in the patch pipette before applying paired conditioning stimulation (Figure 5 and 6). We have previously shown that this peptide can block internalization of EGFP-tagged Kv4.2 as well as endogenous A-type K^+^ channels in cultured neurons [12]. In the presence of DYN, EPSC amplitude was monitored after paired conditioning stimulation and A-currents were recorded pre-LTP (“control” with DYN, n = 10), 10 (n = 6) and 30 min post-LTP (n = 8) after pulling nucleated patches. With DYN in the pipette, EPSC amplitude was significantly increased initially after paired stimulation (Figure 5A; “10 min post-LTP”, 0 min = 77.97±15.93%, p = 0.006). However, this increase in EPSC amplitude progressively diminished such that no potentiation was observed 30 min after paired stimulation (“30 min post-LTP”, 20 min = −1.97±13.61%, p = 0.834), compared with scrambled dynamin (“Paired+sDYN” in Figure 5B). The profile and time course of this potentiation matches well that previously found for neurons overexpressing Kv4.2 or when the calcium/calmodulin-dependent protein kinase II CaMKII is inhibited [20], [21].

Peak amplitude of I_A_ before applying the pairing protocol was not significantly different than that found in patches without intracellular DYN (Figure 5C and D, “Paired+DYN”, n = 10, control, 1.43±0.08 nA, p = 0.538). However, after paired conditioning stimulation DYN blocked the progressive decrease of peak A-current density during potentiation (”Paired+DYN”, 30 min = 1.41±0.08 nA, p = 0.895 compared with “control”), while scrambled DYN still showed a reduction of peak A-current 30 min after paired conditioning (Figure S2 and Figure 5D; “Paired+sDYN”, control = 1.44±0.06, n = 8, 30 min = 0.99±0.06, n = 7, p<0.001).

DYN treatment did not prevent the shift in the voltage-dependence of steady-state A-current inactivation after LTP (Figure 5E–G). The hyperpolarized shift in inactivation curves were observed 10 min after paired conditioning stimulation and returned to the control level by 30 min (V_h_, “Paired+DYN”, control = −61.6±1.4, 10 min = −66.5±0.9, p = 0.021, 30 min = −60.4±0.7 mV, p = 0.489), perhaps indicating a role for the A-current inactivation curve shift in the mechanism of early synaptic potentiation in this preparation.

The effects of the dynamin inhibitory peptide on synaptic potentiation and I_A_ were reflected in its ability to prevent most measures of intrinsic plasticity (Figure 6). After pairing stimulation, we again tested the effect of DYN and scrambled DYN on intrinsic excitability every 10 min (Figure 6B–E). Including the dynamin inhibitory peptide in pipette solution completely blocked the increase of AP firing rate, which was observed in experiments without DYN after paired conditioning stimulation (Figure 6B; “Paired+DYN”, 100 pA current injection, pre = 42.84±4.79, 30 min = 45.01±4.22 Hz, n = 16, p = 0.711). Scrambled DYN did not prevent the increase in firing (“Paired+sDYN”, 100 pA injection, pre = 39.98±3.39, 30 min = 59.39±4.13 Hz, n = 6, p = 0.008). In Figure 6C, we show that DYN also prevented changes in the AHP (Figure 6C; “Paired+DYN”, pre = −7.24±0.87, 30 min = −6.75±1.02 mV, p = 0.727) and AP threshold (Figure 6D; “Paired+DYN”, pre = −36.14±0.59, 30 min = −37.93±0.84 mV, p = 0.108; “Paired+sDYN”, pre = −37.12±0.68, 30 min = −41.80±1.04 mV, p = 0.006). DYN also blocked the change in onset time of the first AP induced by a 100 pA current injection although a trend is still apparent (“Paired+DYN”, pre = 39.78±4.11, 30 min = 30.63±4.01 ms, p = 0.153). The block of LTP by DYN means that endocytosis is a necessary step for the formation of long-lasting synaptic potentiation. Without a specific blocker of Kv4.2 channel internalization, we can not exclude the possibility that DYN is blocking endocytosis of another protein critical to LTP and intrinsic plasticity induction. However, the most parsimonious interpretation of these results would be that the internalization of A-type channels substantially contributes to the enhanced excitability after LTP induction.

**Figure 7:**
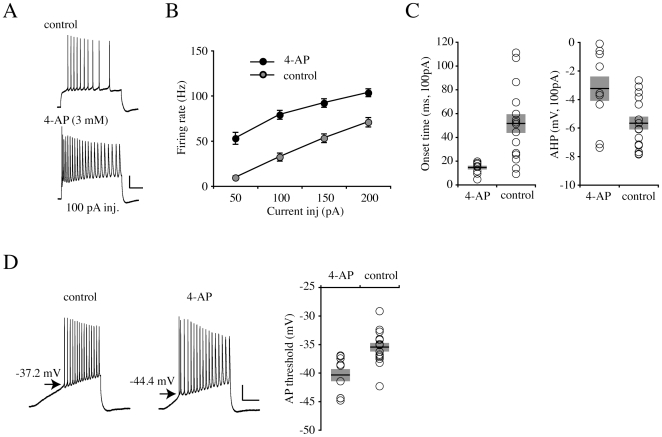
Intracellular 4-AP application increases intrinsic excitability. A. Example traces of AP firing in response to +100 pA injection in recording 15–30 min after whole cell formation without (control) or with 3 mM 4-AP (4-AP) included in the pipette solution. 4-AP increases onset time and frequency. Scale bars: 20 mV, 200 ms. B. Averaged firing rates. After 4-AP washin, neurons showed a significant increase in AP firing rate. Error bars represent SEM. C. Averaged AP onset time and AHP. Both properties were also decreased by 4-AP addition. Bars indicate the mean values and semitransparent boxes represent SEM. D. Example traces of AP firing after injecting ramp currents (250 pA/s). A significant decrease in AP threshold was observed in the presence of 4-AP. Scale bars: 20 mV, 200 ms. Bars indicate the mean values and semitransparent boxes represent SEM.

To confirm that the magnitude of I_A_ is important for the regulation of intrinsic excitability, we added 4-AP (3 mM) to pipette recording solution. We have previously found that this concentration of internal 4-AP can reduce somatic I_A_ by about 60% in nucleated patches and is relatively specific for A-type over sustained K^+^ currents [21]. AP firing patterns and threshold were observed 15–30 min after obtaining whole-cell recordings (Figure 7, “4-AP”, n = 9). AP firing rate was significantly enhanced in neurons experiencing intracellular 4-AP (Figure 7A and B, 100 pA current injection, 4-AP = 78.83±4.91, control = 32.08±4.39 Hz, p<0.001). In addition, both AP onset time and AHP were clearly reduced in 4-AP containing neurons (Figure 7A and C; “Onset time”, 4-AP = 14.57±1.57, control = 51.35±7.87 ms, n = 15, p<0.001; “AHP”, 4-AP = −3.25±0.85, control = −5.67±0.45 mV, p = 0.035). Finally, ramp current injections showed that 4-AP can reduce AP threshold (Figure 7D. 4-AP = −40.41±1.06, control = −35.51±0.75 mV, p = 0.003), similar to our previous results upon extracellular 4-AP application [8].

### Global effect of intrinsic plasticity on dendritic integration

These results presented so far, along with previous results showing an effect of A-type K^+^ channels on synaptic currents [9], [12], [22], [23] (reviewed in [24]), suggests the targeting A-type channels for activity-dependent regulation of intrinsic and synaptic plasticity. However, whereas synaptic plasticity is generally considered to be a local, if not synapse specific phenomenon [25], changes in intrinsic excitability are global, suggesting consequences on the integration of unpotentiated synapses [4] Therefore, we tested the extent of intrinsic plasticity by measuring dendrosomatic coupling of synapses not receiving paired stimulation in neurons receiving another set of potentiated inputs.

**Figure 8:**
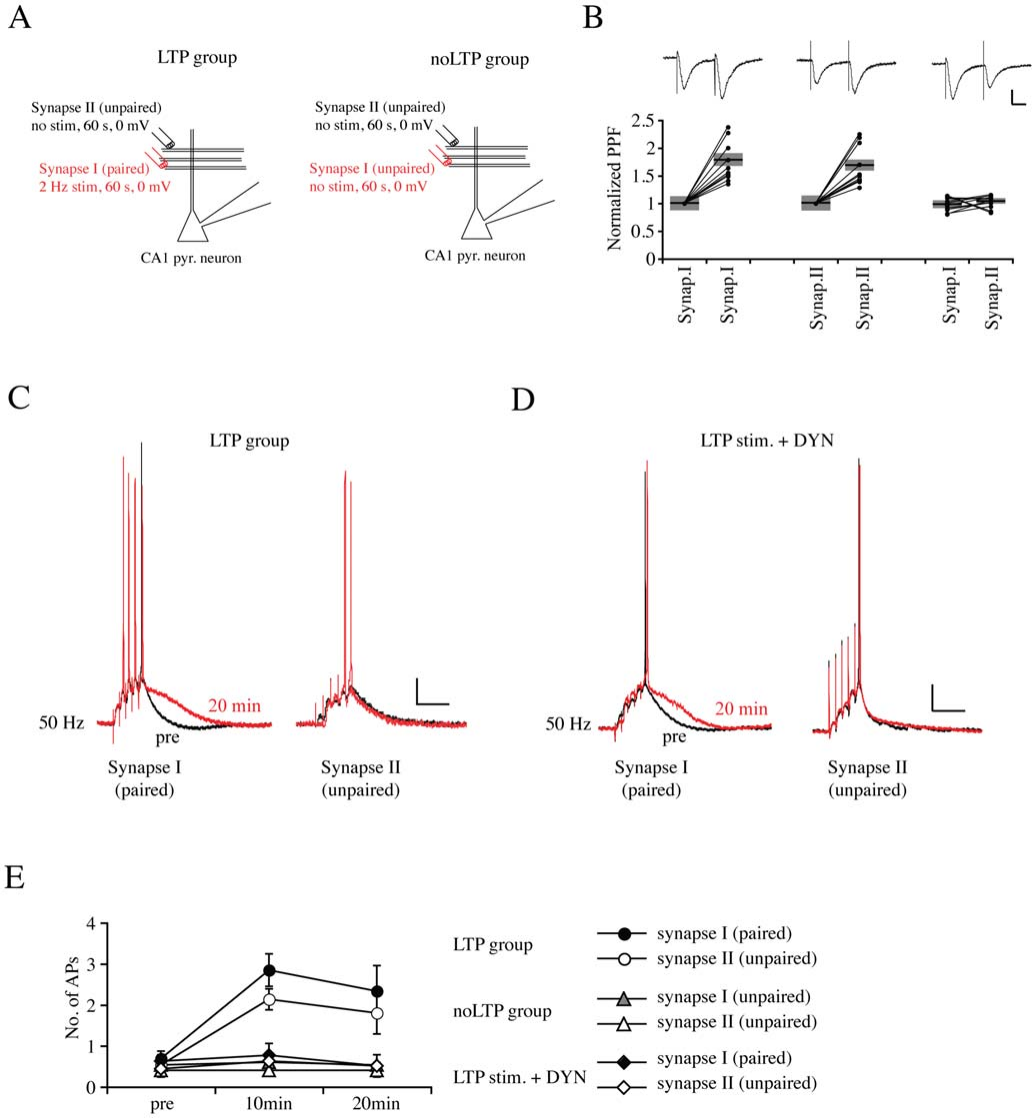
Intrinsic plasticity enhances the throughput of unpotentiated synaptic inputs. A. The experimental protocol used to test the effect of intrinsic plasticity on dendritic signal processing. Two independent synaptic pathways were recorded during each experiment. During conditioning stimulation, a 2 Hz stimulation (for 1 min) was delivered to only one pathway at a 0 mV holding potential (Synapse I (paired) in “LTP” group). For comparison, both sets of synapses did not receive any conditioning stimulation at a 0 mV holding potential for 1 min in some experiments (“noLTP” group). The two pathways in all experiments were separated by 50∼120 µm. All other recording parameters of recording are the same as in Figure 1A. B. A crossed paired-pulse facilitation (PPF) assay was used to confirm pathway independence. Scale bars: 50 pA, 20 ms. Bars indicate the mean value and semitransparent boxes represent SEM. C. Example traces of EPSPs triggered by a train of 5 stimulations at 50 Hz. Before applying conditioning stimulation (black, pre), the stimulation intensity was adjusted to less than threshold. As expected, after conditioning stimulation, EPSP–spike coupling was significantly increased (red traces, 20 min, Synapse I (paired)) in neurons receiving paired stimulation. However, even synapses not exhibiting LTP (Synapse II (unpaired), see also Figure S3C and D) showed increased firing. Scale bars: 10 mV, 100 ms. D. Example traces of EPSPs triggered by a train of 5 stimulations at 50 Hz in the presence of DYN. All recording protocol is as “C”, Scale bars: 10 mV, 100 ms. DYN completely blocked the enhanced EPSP-spike coupling in both pared and unpaired pathways. E. AP firing during a 50 Hz stimulation in neurons receiving paired and unpaired stimulation. After LTP induction both sets of synapses in the “LTP” group showed enhanced AP firing, which was blocked by DYN application (LTP+DYN). Error bars represent SEM.

To investigate the non-specific consequences of intrinsic plasticity, we simultaneously triggered EPSPs from two independent sets of synapses and measured the effect of intrinsic plasticity on EPSP summation and the ability to elicit APs (Figure 8 and Figure S3). In this set of experiments, one pathway (“Synapse I”) received paired stimulation while the second pathway (“Synapse II”) received only depolarization (Figure 8A, “LTP group”). After having first confirmed pathway independence using a crossed paired-pulse facilitation (PPF, Figure 8B) test, synapse-specific plasticity was monitored. In another experimental group, for comparison, neither pathway received paired stimulation (Figure 8A, “noLTP group”). Results showed that only pathways receiving paired conditioning stimulation showed LTP (Figure S3A). Before and after LTP induction, we measured EPSP summation in both pathways every 10 min (Figure 8C and E). Each test stimulation consisted of 5 pulses at 50 Hz. As expected, in the paired pathway, the probability of AP firing by EPSP trains was significantly increased 10 and 20 min after conditioning stimulation (Figure 8C and E; “LTP group–Synapse I”, pre = 0.69±0.17, 10 min = 2.84±0.40, n = 9, p = 0.013 compared with “noLTP group”, n = 5). However, enhanced EPSP-AP coupling was also observed in the unpaired pathway that did not show synaptic potentiation (Figure 8C and E; “LTP group–Synapse II” pre = 0.53±0.15, 10 min = 2.13±0.26, p = 0.004). The enhanced probability of AP firing in unpaired pathways of neurons showing LTP at other synapses was also observed at other stimulation frequencies (data not shown, 20 Hz; “Synapse I (paired)”, pre = 0.67±0.21, 10 min = 2.53±0.41, p = 0.003; “Synapse II (unpaired)”, pre = 0.53±0.16, 10 min = 2.67±0.43, p = 0.001, data not shown). Consistent with the effect of DYN on intrinsic plasticity and synaptic LTP, global enhanced dendrosomatic coupling was completely blocked in neurons exposed to intracellular DYN (Figure 8D and E, “LTP+DYN”, n = 7, 10 min, paired = 0.77±0.29, unpaired = 0.63±0.28). These findings indicate a critical role for the dynamic regulation of A-type channels in regulating neuronal output.

## Discussion

We report here a two-phased decrease in A-type K^+^ channel activity that contributes to the plasticity of intrinsic excitability induced by synaptic LTP in CA1 pyramidal neurons of hippocampal organotypic slice cultures. Initially, within ten minutes of LTP induction, we found a hyperpolarized shift in the voltage-dependence of steady-state inactivation curve for A-channels in recordings from nucleated patches as was previously shown to enhance dendritic excitability after LTP [18]. This curve shift was temporary however; showing no difference from pre-LTP values 20 min after LTP induction. The second phase consisted of a slow, progressive loss of peak A-current density after LTP. The time-course of this reduction matched that found previously for activity-dependent internalization of A-type currents [12] and blocking clathrin-mediated endocytosis by a dynamin-based inhibitory peptide prevented the expression of synaptic and intrinsic plasticity. These findings suggest that these two temporally distinct but overlapping mechanisms of A-channel down-regulation together contribute to enhance intrinsic excitability and strengthened EPSP-AP coupling observed in CA1 pyramidal neurons after the induction of synaptic plasticity; including enhanced responsiveness to unpotentiated synapses. Importantly, no change in excitability was found in neurons receiving depolarization but not stimulated during the depolarization (Figure 8 and Supplemental Figures 3 and 5), demonstrating the requirement of physiological (i.e. synaptic) activity for intrinsic plasticity.

### LTP induced A-type K^+^ current down-regulation and intrinsic plasticity

In our recordings of K^+^ currents from nucleated patches, a hyperpolarizing shift in the voltage-dependence of steady-state inactivation resulted in an ∼60% decrease in peak I_A_ measured from a holding potential of −60 mV (the approximate resting membrane potential of these neurons, Figure 3). A similar inactivation curve shift was previously reported as a main modulatory factor for local increases in dendritic excitability during synaptic potentiation [18]. As both local dendritic and somatic membrane excitability are regulated by A-type channels [8], [18] this immediate but short-lasting reduction of I_A_ via inactivation curve shift during the initial period of potentiation can induce a rapid, significant enhancement of excitability throughout the neuron. Enhanced excitability may then directly affect the properties and/or expression of other ion channels, affecting membrane conductance (for review, see Reyes 2001[26]) and/or impact cellular signaling cascades.

This initial and rapid decrease in I_A_ is not, however, sufficient to maintain long-term intrinsic plasticity as the inactivation curve shifted back to control levels by 20 min after synaptic LTP induction. Furthermore, blocking clathrin-mediated endocytosis with DYN (a dynamin-based inhibitory peptide) prevented most measures of intrinsic plasticity as well as synaptic potentiation beyond 20 min (Figure 5 and 6). This peptide has been shown previously to block activity-dependent internalization of EGFP-tagged Kv4.2 and prevent the activity-dependent reduction of endogenous somatic A-type currents [12]. In Kim et al. 2007, we also reported that blocking endocytosis significantly reduced membrane depolarization during depolarizing global AMPA stimulation of dissociated hippocampal neurons. In the present study, NMDAR-dependent synaptic LTP induction lead to a progressive decrease of peak I_A_, which was completely blocked by intracellular application of DYN (Figure 4 and 5). That the peptide did not interfere with the initial inactivation curve shift indicates a larger role for internalization of A-type channels in the expression of intrinsic plasticity, impacting synaptic throughput of both potentiated and naive synapses. Further research will be needed to investigate the mechanism behind the hyperpolarizing shift in the A-channel inactivation curve and to determine if it acts as a trigger for the second phase of I_A_ down-regulation and/or the induction/early phase of synaptic plasticity, which is spared in experiments including intracellular DYN.

### Alternative mechanisms for intrinsic plasticity

Although our results suggest I_A_ regulation as a primary factor in intrinsic plasticity, other mechanisms may also contribute, as preventing endocytosis did appear to completely abolish the pairing-induced acceleration of AP onset time (Figure 6). In addition, a previous report from juvenile rat acute slice recordings of CA1 neurons found that the activation curve of voltage-gated Na^+^ channels is hyperpolarized after LTP, acting to increase excitability [13]. However, the same study also found Na^+^ channel inactivation curve to be hyperpolarized after LTP, decreasing the number of Na^+^ channels available for activation, which would be expected to decrease excitability. In our preparation, we found a slight but not significant *decrease* in the maximal AP rate of rise to decrease in both paired and unpaired recordings indicating, if anything, a decrease in Na^+^ channel efficacy with LTP (Figure S4A and B).

In another previous report, LTP induced by theta burst pairing was reported to depress neuronal excitability through the upregulation of hyperpolarization-activated (I_h_) channels in adult hippocampal CA1 neurons [16]. Subsequently Campanac et al., 2008 determined that I_h_ could be up- or down-regulated after LTP depending on the method of induction and resulting strength of potentiation. In our preparation, we observed an increase of I_h_, as assessed by the depolarizing sag induced during negative current injections (−150 to −50 pA), in recordings of paired neurons (Figure S4C). The increase in I_h_ was not accompanied by a significant change in input resistance (R_in_) in paired recordings (50 min = 105±7%, n = 6, p = 0.428 compared with “pre”, n = 6). The effect of increasing Ih on R_in_ was presumably offset by loss of A-type current which would enhance R_in_ [8].

The enhanced I_h_ after LTP was not blocked in DYN experiencing neurons, which showed neither synaptic nor intrinsic plasticity (Figure 8D, “Synapse I (paired)” and Figure S4C “Paired+DYN”). These findings indicate that dynamic regulation of I_h_, at least at this developmental stage, does not effectively contribute to intrinsic plasticity in the absence of endocytosis. In rat brains, functional expression and activation of I_h_ channels are age-dependent [27], so their modulation may be more effective in regulating excitability in neurons from older rats [3], [16].

### Effect of intrinsic plasticity on EPSPs summation of unpaired synapses

Our finding that potentiation of intrinsic excitability significantly facilitated the EPSP-spike coupling of unpaired synapses as well as paired synapses (Figure 8) indicates that the potentiation of intrinsic excitability plays a powerful role in synaptic integration, enhancing synaptic gain globally. In our experiments, paired and unpaired synapses were separated by at least 50 µm. With this distance of separation, LTP should be synapse specific [25] and synaptic plasticity clearly occurred in a synapse-specific manner, as paired but not unpaired synapses showed enhanced EPSC amplitude in the LTP group (Figure S3). Enhanced EPSP-spike coupling of both paired and unpaired synapses was not observed in neurons treated with DYN, despite larger EPSPs in the pairing condition. Enhanced I_h_, reducing EPSP summation, may account for this observation but in general this result suggests a powerful role for somatic channels in determining neuronal output (Figure 8).

### Intrinsic plasticity as a mechanism for memory storage

A long-lasting increase of EPSP-AP coupling in CA1 neurons has been put forward as a memory-storage mechanism (for reviews see [4], [5]). Increases in intrinsic excitability are observed after training in behavior learning tasks in vertebrate as well as invertebrate animals, with the targeting of K^+^ channels for downregulation being a commonly observed mechanism. Our results show a decreased AP threshold, which would seem to be a principal target for cell-wide synaptic gain modulation. Our finding that unpaired synapses can become more highly coupled to AP initiation suggests the possibility that synaptic plasticity and associated changes in dendritic processing may act to trigger changes in intrinsic excitability under some learning paradigms. Previously, we reported activity-dependent internalization of Kv4.2 channels from spines after inducing chemical LTP [12]. As the potentiation of synaptic strength increases the local dendritic excitability and subsequent Ca^2+^ influx [18], the expression level of I_A_ in dendrites and synaptic sites may then be regulated by neurons to initiate secondary computational processing throughout the neuron. Regardless of the molecular mechanisms involved, this role of synaptic potentiation as a trigger for intrinsic plasticity does not preclude a role for synapse specific mechanisms of plasticity in other types of memory storage [28].

## Materials and Methods

### Tissue preparation

Organotypic hippocampal slices (350 µm thick) were prepared from postnatal day 7–8 Sprague-Dawley rats. After preparing hippocampal slices in a cutting solution containing (in mM): 10 MgCl_2_, 25 Glucose, 20 Hepes in Gey's balanced salt solution (Sigma), slices were transferred to an incubator gassed with 95% O_2_ and 5% CO_2_ at 35°C, and cultured for 5–6 days before performing electrophysiological measurements. More detailed protocols for tissue preparation and recording techniques are available in previous papers [8], [12], [21]. The National Institute of Child Health and Human Development's Animal Care and Use Committee approved all animal protocols.

### Electrophysiology

For patch-clamp recordings from organotypic slices, slices were transferred to a submerged recording chamber with continuous flow of ACSF containing (in mM): 125 NaCl, 2.5 KCl, 25 NaHCO_3_, 1.25 NaH_2_PO_4_, 25 Glucose, 2 CaCl_2_, 1 MgCl_2_. 5 µM 2-chloroadenosine and 5 µM bicuculline were added in all recordings. One µM TTX was added to record voltage-dependent K^+^ currents immediately after pulling nucleated patches. Patch electrodes (4–6 MΩ) were filled with (in mM): 20 KCl, 125 Kglu, 10 HEPES, 4 NaCl, 0.5 EGTA, 4 ATP, 0.3 TrisGTP and 10 Phosphocreatin. pH and osmolarity were adjusted to 7.2–7.3 and 280–300 mOsm, respectively, in all experiments. No corrections were made for liquid junction potentials. In whole cell recordings, EPSCs and EPSPs were induced by stimulation of the Schaffer-collateral pathway via bipolar electrodes located ∼150 µm from the soma of the recorded cell. The test stimulation to elicit EPSCs in LTP experiments was set at 0.1 Hz with 0.2 ms duration. Stimulation intensity (100–900 µA) was adjusted to induce ∼−100 pA EPSCs (−60 mV holding potential). APs to detect changes in intrinsic excitability were induced by a series of current injections in current-clamp mode. A 5-pulse train at 10, 20 and 50 Hz was used to test EPSP summation and AP initiation. Whole-cell recording parameters were monitored throughout each experiment and recordings where series resistance (9–22 Mohm) varied by more than 10% were rejected. DYN and sDYN peptides were prepared and used as in Kim et al., 2007. Peptides were intracellularly perfused into the cell for 20–25 min before recording.

In this study, synaptic LTP was induced by a pairing protocol, which consisted of low frequency stimulation (2 Hz, 0.2 ms duration) paired with depolarization to 0 mV for 1 min. Either low frequency stimulation without depolarization to 0 mV (n = 27) or depolarization to 0 mV for 1 min without stimulation (n = 5) was used as an unpaired group for control experiments. Potentiation was monitored for 30–50 min post-induction.

All recordings were performed at 31–32°C and low-pass filtered at 5 kHz and digitized at 10 kHz by an Instrutech ITC-18 A/D board controlled by software written for Igor Pro (WaveMetrics). An Axopatch-200B amplifier was employed for whole-cell patch recordings in this study. Command pulse generation, data acquisition and analysis were performed using IGOR Pro (Wavemetrics). Excel (Microsoft) software was used for further data and statistical analysis. Statistical tests performed were unpaired t-tests and significance was set to p<0.05.

Nucleate patch recordings of voltage-gated K^+^ currents were made in voltage-clamp mode (21.75+0.52 pF, n = 88). Current ensemble averages were constructed from 3 individual sweeps. Leakage and capacitive currents were subtracted digitally using either a P/5 protocol or null traces. The transient current was isolated from the sustained current using 200 ms prepulse step to −20 mV to inactivate transient channels. All curve fits (inactivation time constants, Boltzmann fits and various x-y plots) were performed with a least-squares program (Igor Pro).

## Supporting Information

Figure S1 Nucleated patches (arrows) were formed 10, 20 or 30 min after conditioning stimulations. Before making the nucleated patch, synaptic current amplitudes were monitored in all experiments. Inserted picture shows a nucleated patch. Numbers in parentheses represent the number of patches for each time point. Error bars represent SEM.
(4.59 MB TIF)

Figure S2 A. Example traces of peak transient and sustained currents before (pre-LTP) and 30 min after LTP (30 min post-LTP) in the presence of scrambled DYN (sDYN). Peak amplitude of IA was significantly decreased 30 min after LTP induction. Recording protocol is as in Figure 4A. Averaged values are shown in Figure 5D. Scale bars: 200 pA, 100 ms. B. Example traces used to construct steady-state inactivation curves. Red traces are transient currents recorded for a −60 to +60 mV step. Recording protocol is as Figure 3A. Scale bars: 200 pA, 100 ms. C. Pooled data showing the normalized voltage-dependence of activation and inactivation before (control) and 30 min after LTP induction in the presence of scrambled DYN. No change was observed after LTP. Error bars represent SEM.
(7.63 MB TIF)

Figure S3 Synaptic LTP by pairing protocol shows the synapse-specificity. A. Example traces of EPSCs after synaptic potentiation in the “LTP” group. Only EPSCs from the paired pathway was facilitated after conditioning stimulation. Scale bars: 50 pA, 20 ms. Pooled LTP data from paired and unpaired synapses (lower panel). Arrows indicate times where EPSP summation was measured (Figure 8). Error bars represent SEM. B. LTP patterns in the presence of DYN. All recording procedures are as in “A”. Scale bars: 10 pA, 20 ms. Pooled LTP data are shown in lower panel. Error bars represent SEM.
(7.66 MB TIF)

Figure S4 LTP induces no changes of AP max. rising rate but increases Ih current during LTP. A. Example traces of AP firing before (pre) and 30 min after LTP induction. Overlaid are the 1st temporal derivatives showing no changes in the peak rate of rise after LTP induction. Traces in the right show the 1st AP aligned with its derivative. The bottom set of traces show the overlaid 1st derivatives before (red) and after (green) LTP induction. Scale bars: 100 mVs-1, 2 ms. B. Pooled data showing not significant in the rate of rise before and after LTP induction in paired and unpaired neurons. Error bars represents SEM. C. Ih component induced by negative current injections (−150∼−50 pA with 50 pA step), was significantly increased in paired neurons (Paired). There was no significant change in input resistance in either the paired or unpaired recordings using methods described in Kim et al., 2005. Interestingly, this enhancement of Ih component was observed in neurons treated with DYN (Paired+DYN), which did not show synaptic potentiation. “Sag” indicates the difference between “Peak” and “Steady-state” voltages. Scale bars: 10 mV, 200 ms, Scale bars: 10 mV, 200 ms. Error bars represent SEM.
(0.90 MB TIF)

Figure S5 No change of excitability in CA1 neurons by depolarization alone. To test if depolarization alone can induce the changes in CA1 excitability, neurons were held at a 0 mV holding potential for 1 min without pairing stimulation. This depolarizing condition did not induce any changes of parameters to indicate the excitability of CA1 neurons or Ih currents. A. Example traces before (pre, black) and 10 min after (post, red) depolarization. The recording protocol is as in Figure 2. Scale bars: 20 mV, 200 ms. B. Pooled data showing no significant changes in AP firing properties and threshold before and after depolarization. C. Ih induced by negative current injections (−150 to −50 pA in 50 pA increments), was not changed before (pre, black) and after (post, red) depolarization. Scale bar: 20 mV, 200 ms. Error bars represent SEM.
(0.64 MB TIF)
