# Supplementary figures and images for: Biphasic Somatic A-Type K+ Channel Downregulation Mediates Intrinsic Plasticity in Hippocampal CA1 Pyramidal Neurons
Source: PLoS One. 2009 Aug 7;4(8):e6549. doi: 10.1371/journal.pone.0006549 (PMC2717216; doi:10.1371/journal.pone.0006549)

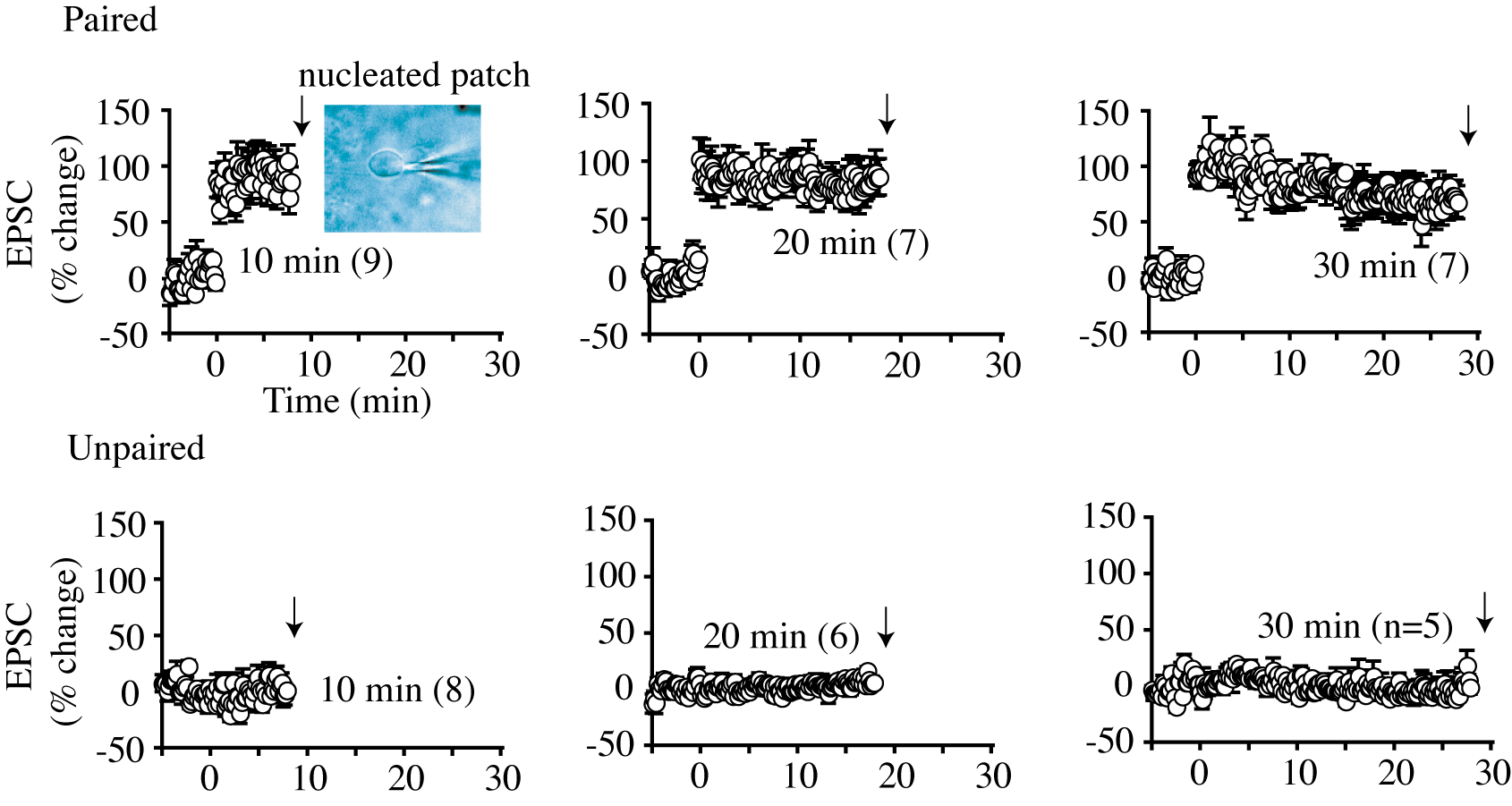

Supplement: Figure S1 — Nucleated patches (arrows) were formed 10, 20 or 30 min after conditioning stimulations. Before making the nucleated patch, synaptic current amplitudes were monitored in all experiments. Inserted picture shows a nucleated patch. Numbers in parentheses represent the number of patches for each time point. Error bars represent SEM. (4.59 MB TIF) [file pone.0006549.s001.tif]

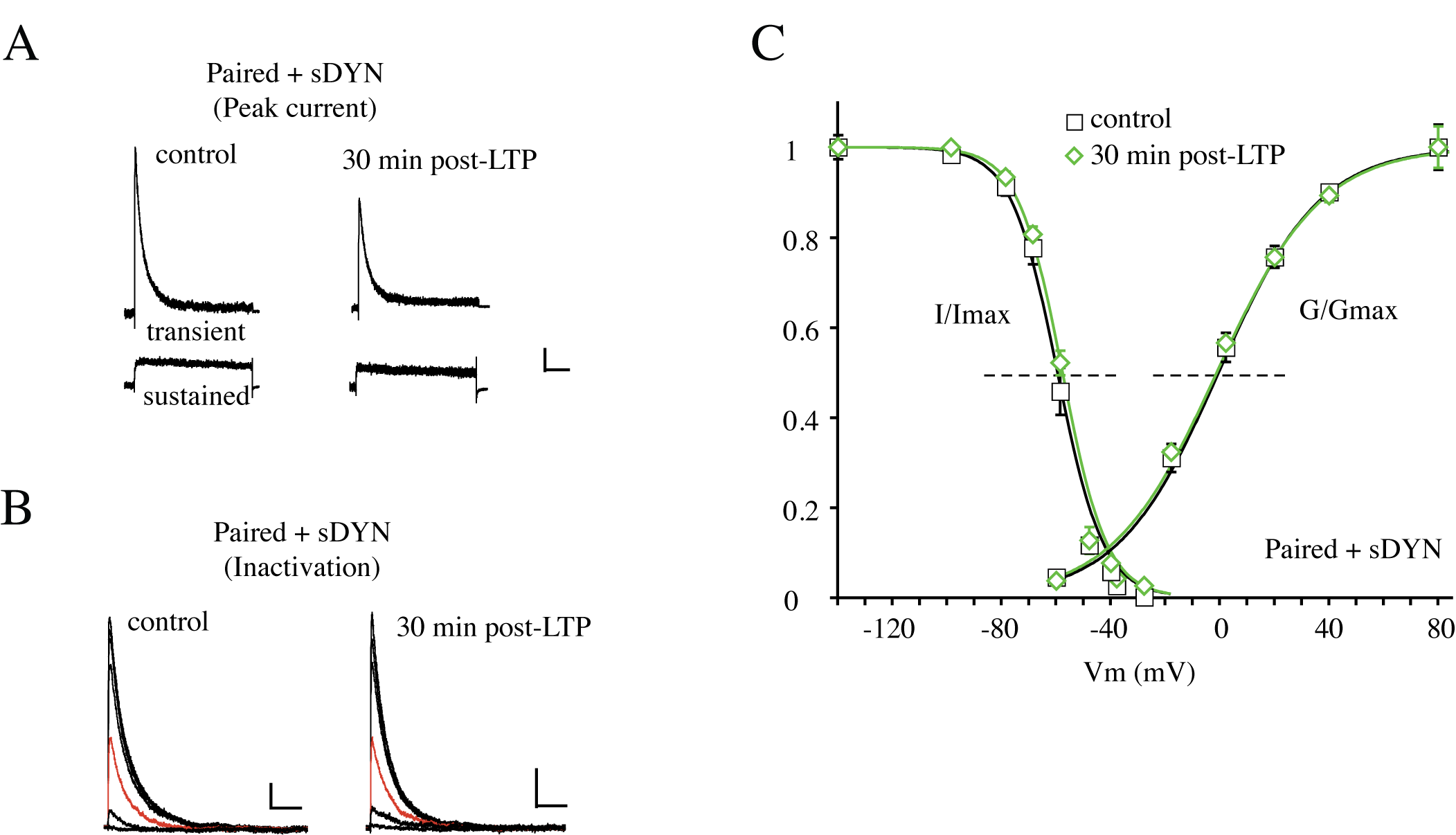

Supplement: Figure S2 — A. Example traces of peak transient and sustained currents before (pre-LTP) and 30 min after LTP (30 min post-LTP) in the presence of scrambled DYN (sDYN). Peak amplitude of IA was significantly decreased 30 min after LTP induction. Recording protocol is as in Figure 4A. Averaged values are shown in Figure 5D. Scale bars: 200 pA, 100 ms. B. Example traces used to construct steady-state inactivation curves. Red traces are transient currents recorded for a −60 to +60 mV step. Recording protocol is as Figure 3A. Scale bars: 200 pA, 100 ms. C. Pooled data showing the normalized voltage-dependence of activation and inactivation before (control) and 30 min after LTP induction in the presence of scrambled DYN. No change was observed after LTP. Error bars represent SEM. (7.63 MB TIF) [file pone.0006549.s002.tif]

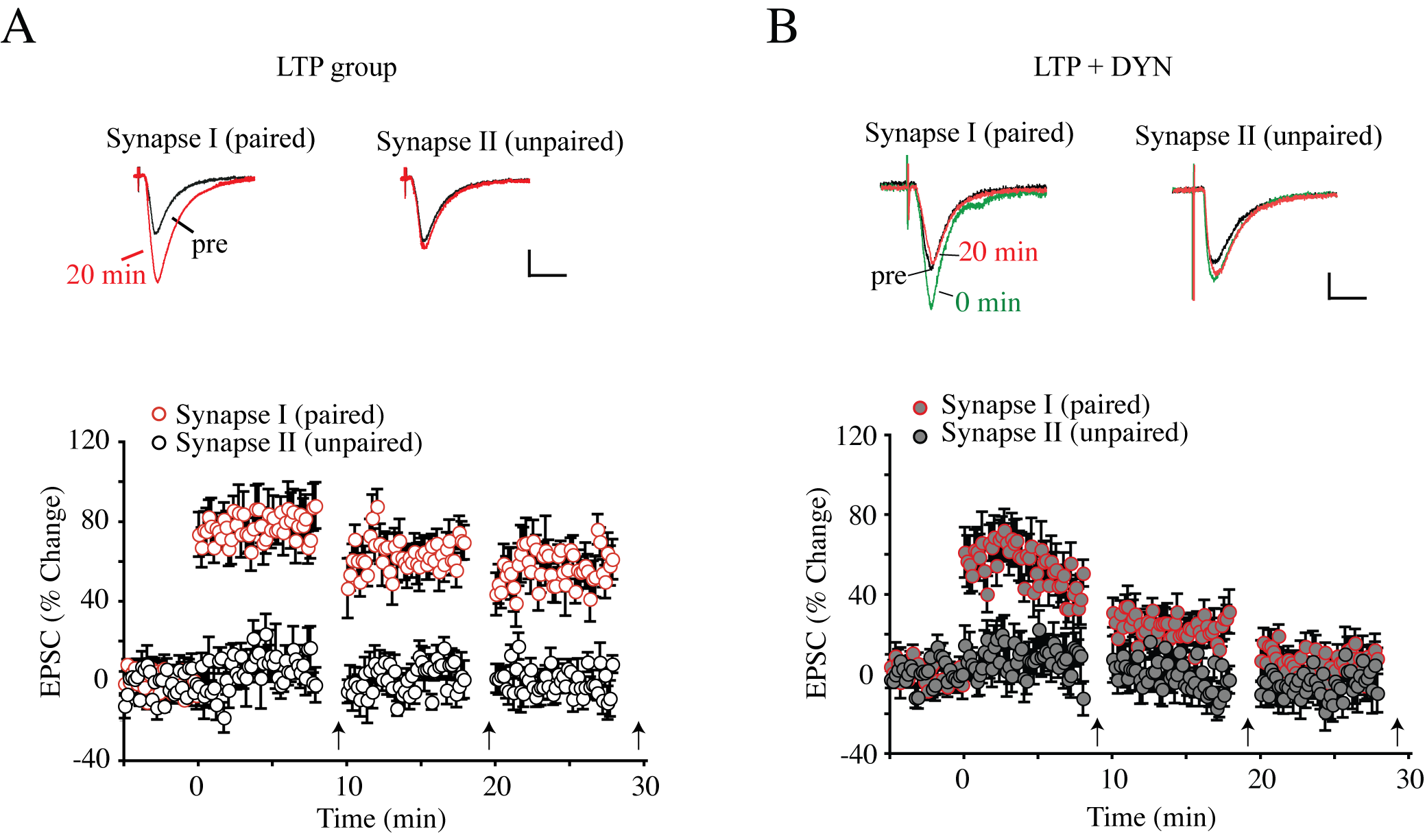

Supplement: Figure S3 — Synaptic LTP by pairing protocol shows the synapse-specificity. A. Example traces of EPSCs after synaptic potentiation in the “LTP” group. Only EPSCs from the paired pathway was facilitated after conditioning stimulation. Scale bars: 50 pA, 20 ms. Pooled LTP data from paired and unpaired synapses (lower panel). Arrows indicate times where EPSP summation was measured (Figure 8). Error bars represent SEM. B. LTP patterns in the presence of DYN. All recording procedures are as in “A”. Scale bars: 10 pA, 20 ms. Pooled LTP data are shown in lower panel. Error bars represent SEM. (7.66 MB TIF) [file pone.0006549.s003.tif]

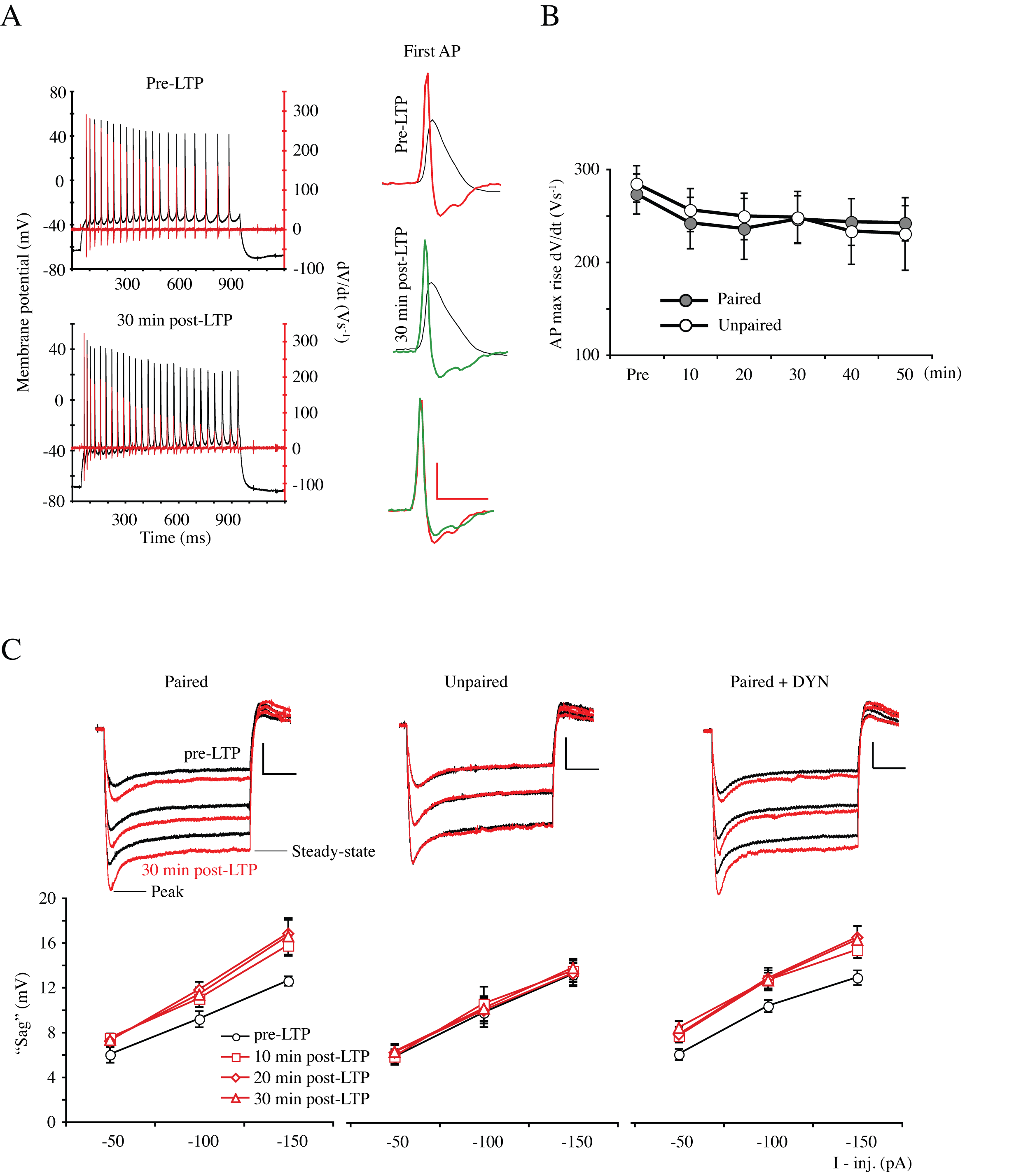

Supplement: Figure S4 — LTP induces no changes of AP max. rising rate but increases Ih current during LTP. A. Example traces of AP firing before (pre) and 30 min after LTP induction. Overlaid are the 1st temporal derivatives showing no changes in the peak rate of rise after LTP induction. Traces in the right show the 1st AP aligned with its derivative. The bottom set of traces show the overlaid 1st derivatives before (red) and after (green) LTP induction. Scale bars: 100 mVs-1, 2 ms. B. Pooled data showing not significant in the rate of rise before and after LTP induction in paired and unpaired neurons. Error bars represents SEM. C. Ih component induced by negative current injections (−150∼−50 pA with 50 pA step), was significantly increased in paired neurons (Paired). There was no significant change in input resistance in either the paired or unpaired recordings using methods described in Kim et al., 2005. Interestingly, this enhancement of Ih component was observed in neurons treated with DYN (Paired+DYN), which did not show synaptic potentiation. “Sag” indicates the difference between “Peak” and “Steady-state” voltages. Scale bars: 10 mV, 200 ms, Scale bars: 10 mV, 200 ms. Error bars represent SEM. (0.90 MB TIF) [file pone.0006549.s004.tif]

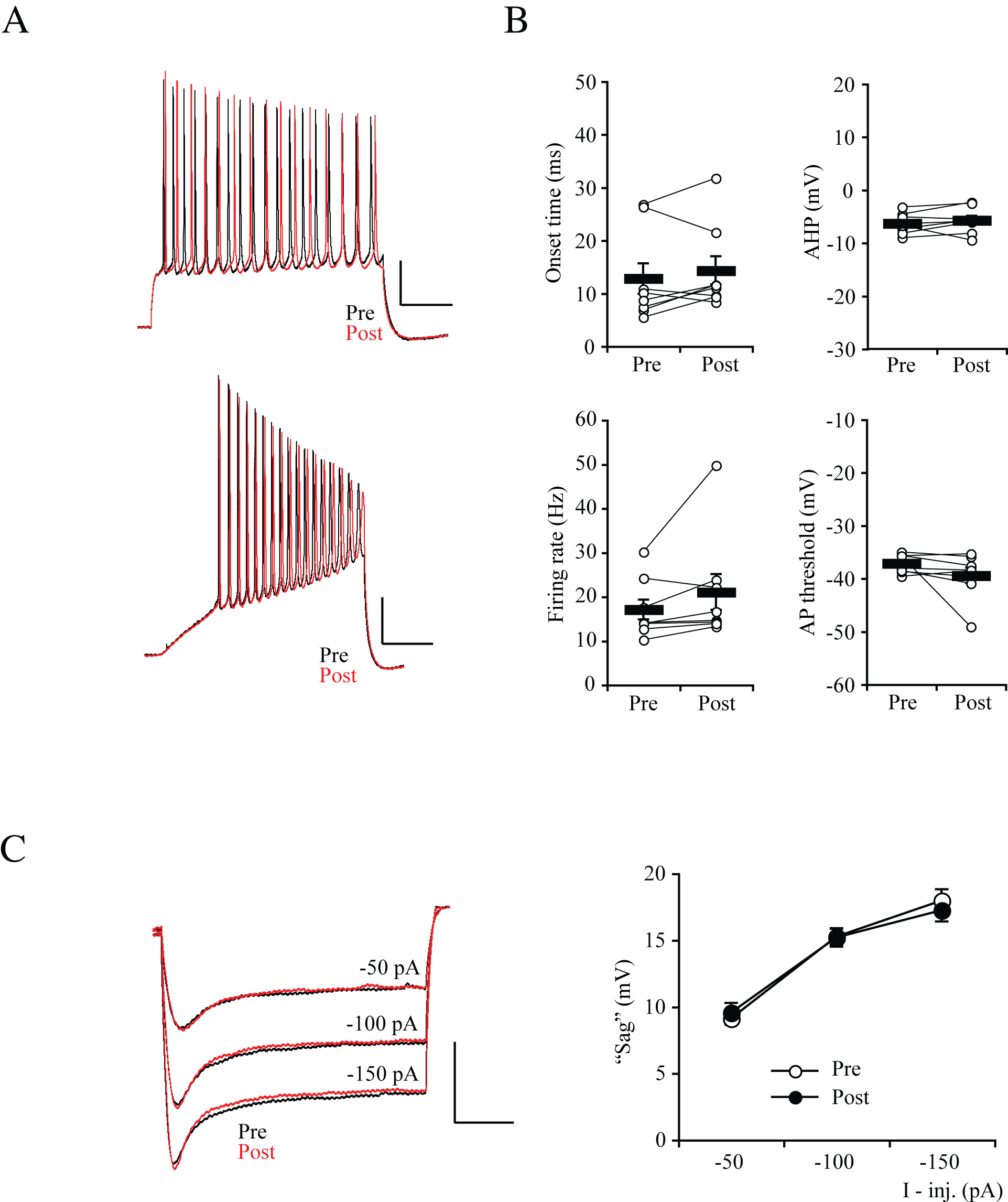

Supplement: Figure S5 — No change of excitability in CA1 neurons by depolarization alone. To test if depolarization alone can induce the changes in CA1 excitability, neurons were held at a 0 mV holding potential for 1 min without pairing stimulation. This depolarizing condition did not induce any changes of parameters to indicate the excitability of CA1 neurons or Ih currents. A. Example traces before (pre, black) and 10 min after (post, red) depolarization. The recording protocol is as in Figure 2. Scale bars: 20 mV, 200 ms. B. Pooled data showing no significant changes in AP firing properties and threshold before and after depolarization. C. Ih induced by negative current injections (−150 to −50 pA in 50 pA increments), was not changed before (pre, black) and after (post, red) depolarization. Scale bar: 20 mV, 200 ms. Error bars represent SEM. (0.64 MB TIF) [file pone.0006549.s005.tif]
